# Pulsatile reverse flow actuated microfluidic injector: toward the application for single-molecule chemotropism assay†

**DOI:** 10.1039/d1ra04505a

**Published:** 2021-08-09

**Authors:** Naoki Yanagisawa, Elena Kozgunova, Tetsuya Higashiyama

**Affiliations:** Institute of Transformative Bio-Molecules (ITbM), Nagoya University Japan ny@nagoya-u.jp; Division of Biological Science, Graduate School of Science, Nagoya University Japan; Department of Biological Sciences, Graduate School of Science, The University of Tokyo Japan

## Abstract

Microfluidic sample plug injection often relies on electrokinetic means which restrict the application of this technology to biological studies involving living cells. Here, we present a microfluidic injector device that is coupled with a cell culture chamber for chemotropic pollen tubes in order to study the directional cellular growth triggered by ligand–receptor interactions at single molecule resolution. In the reported device, unidirectional fluidic flow is introduced by a syringe pump, and a temporal change in the flow direction that is required for a sample plug injection is realized by operating an on-chip electro-osmotic pump that generates pulsatile reverse flow. A fraction of an injected sample plug can be transferred to the cell culture chamber in a controlled manner, which potentially allows us to study the cellular responses to an injected ligand at high spatiotemporal resolution.

## Abstract 2

A localized chemical delivery technique to study cellular responses to signaling molecules.

## Graphical abstract

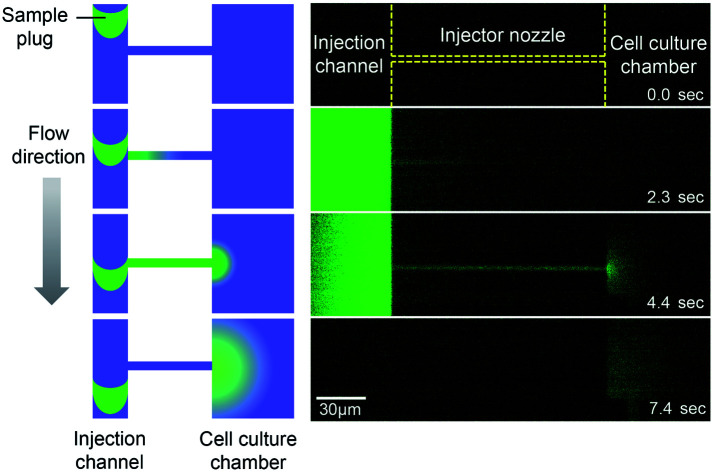

## Introduction

**Fig. 1:**
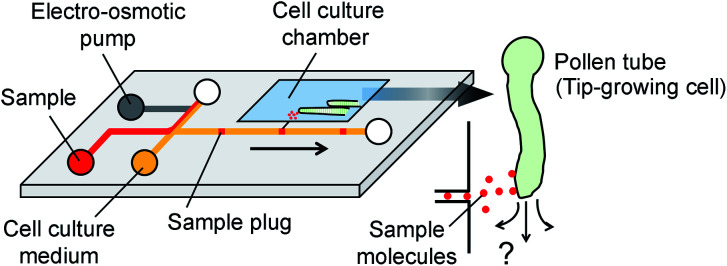
Concept of microfluidic injector for localized chemical delivery on a tip-growing pollen tube. A sample plug is injected by the operation of an on-chip electro-osmotic pump. A fraction of the injected sample plug is transferred to the cell culture chamber where pollen tubes are growing.

Various living cells, such as axons,^1^ fungi,^2^ and pollen tubes,^3^ respond to a gradient of diffusible substances and exhibit distinct directional cell growth, which is referred to as chemotropism. In plant cells, a pollen tube is known to elongate its cell body and to be guided to ovules for fertilization by sensing multiple signaling molecules.^4–7^ Pollen tube chemotropism assays are employed to identify the effects of particular substances on the directional cell growth. Typically, a reagent is embedded in a small bead, which is applied to the apex of a growing pollen tube with the aid of a micromanipulator. While this is a well-established method, it requires a micromanipulator system and an operator who has expertise in its use. In addition, the reagent must be applied to each cell manually, which prevents the possibility of high-throughput operations in this approach. Application of microfluidic devices for plant cell studies have received considerable attention from the plant science community.^8^ Microfluidic sample plug injection techniques, such as pinched injection^9^ or gated injection,^10^ have also been extensively studied but mainly used for conducting analytical separations. Considering their ability to precisely control reagent volumes and injection timings, microfluidic injection techniques can potentially expand their application to any studies that require the supply of a defined volume of a reagent. However, widely used electrokinetic injection methods are not desirable for studies that involve living cells due to their exposure to electric fields. Therefore, all the reported microfluidic devices for pollen tube chemotropism assays are performed under static^11,12^ or pressure-driven flow^13^ (PDF) conditions. While pressure gradients can also be generated on-chip by integrating electro-osmotic (EO) pumps into the device,^14^ dynamic PDF control in microchannels through the operation of EO pumps is challenging or not even feasible when physiologically relevant cell culture medium (*e.g.*, highly conductive liquid medium) is used for the experiments. This is due to the fact that EO pumps rely on the generation of electro-osmotic flow (EOF), and the effective thickness of the electrical double layer formed around the microchannel wall decreases with the use of higher ionic concentration,^15^ resulting in lower EOF velocity (*V*_EOF_) generated under the constant electric field (*E*) as described by:^16^1
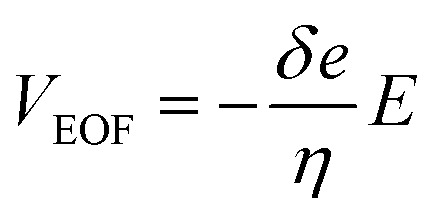
where *δ* is the thickness of the diffuse layer, *e* is the electrical charge per unit surface area, and *η* is the viscosity of the liquid medium. Alternatively, EO pumps have been prepared separately and externally connected to the microdevice.^17^ This approach, however, is limited to the continuous supply of reagents into a cell culture chamber and does not allow localized chemical stimulation at a tip of a growing pollen tube. To address these issues, we have developed a microfluidic sample plug injection technique which is operable under any cell culture medium backgrounds. In our approach, a syringe pump was used to introduce liquid medium into the microfluidic network. Then, a sample plug injection was performed by altering the flow direction with pulsatile reverse flow generated through the operation of an on-chip EO pump. In the reported device, a fraction of a sample plug can be transferred to the integrated pollen tube culture chamber as illustrated in Fig. 1, which enables localized chemical stimulation at a tip of a growing pollen tube. We have also demonstrated the compatibility of the reported device with a total internal reflection fluorescence microscopy (TIRFM), thereby providing the possibility of studying directional cell growth in response to ligand–receptor interactions with single molecules resolution.

## Experimental

### Preparation of the PDMS microfluidic device

A silicon mold used for preparing the polydimethylsiloxane (PDMS) device was fabricated through a standard photolithography technique. Negative photoresists (SU-8 3005, 3010, and 3025; Microchem Corp.) were spin-coated on a silicon wafer to create desired layer thickness (4 μm, 8 μm, 15 μm, and 20 μm). Maskless photolithography system (DL-1000; Nano System Solutions, Inc.) was used to prepare such multi-photoresist layers. The depths of each microchannel segment are described in Fig. S1.† The PDMS device was prepared by casting a mixture of PDMS (Sylgard 184; Dow Corning) and curing agent in a 10 : 1 weight ratio onto the mold. After the mold was degassed for 30 min in a vacuum chamber, the PDMS layer was cured at 65 °C for 90 min.

### Preparation of the on-chip electro-osmotic (EO) pump

Both the PDMS layer and a glass cover slip (Matsunami, No. 1, thickness: 0.12–0.17) were plasma treated in air using a plasma cleaner (YHS-G, SAKIGAKE-Semiconductor Co., Ltd) to enhance the hydrophilicity of the microchannels. During this process, the side channels in the EO pump were selectively protected from the plasma exposure with a SU-8 photoresist mask (Fig. S2†). After the PDMS and glass substrate were bonded to each other, the device was first degassed in a vacuum chamber for 15 min. This process was important in order to fill all the channel networks with liquid medium without having air bubbles at later steps. Then, 3% (w/v) of ultra-low melting point agarose (Type IX-A; Sigma-Aldrich) dissolved in deionized (DI) water was introduced into the side reservoirs in the EO pump. Since the side channels connected to these reservoirs were covered with the photoresist mask during the plasma treatment, the PDMS surface remained hydrophobic in these sections. In this condition, while the agarose gel was still slowly dragged into the channels by capillary force, it completely stopped at the shallow regions prepared at the ends of the side channels. Then, the side channels were selectively blocked by agarose when the device was cooled at 4 °C for 3 min. Afterward, the remaining EO pump channels were filled with DI water, whereas the rest of the microfluidic network was filled with phosphate buffer saline (1× PBS, pH 7.4) or cell (*Arabidopsis thaliana* pollen tube) culture medium.^6^

### Sample plug injection

**Fig. 2:**
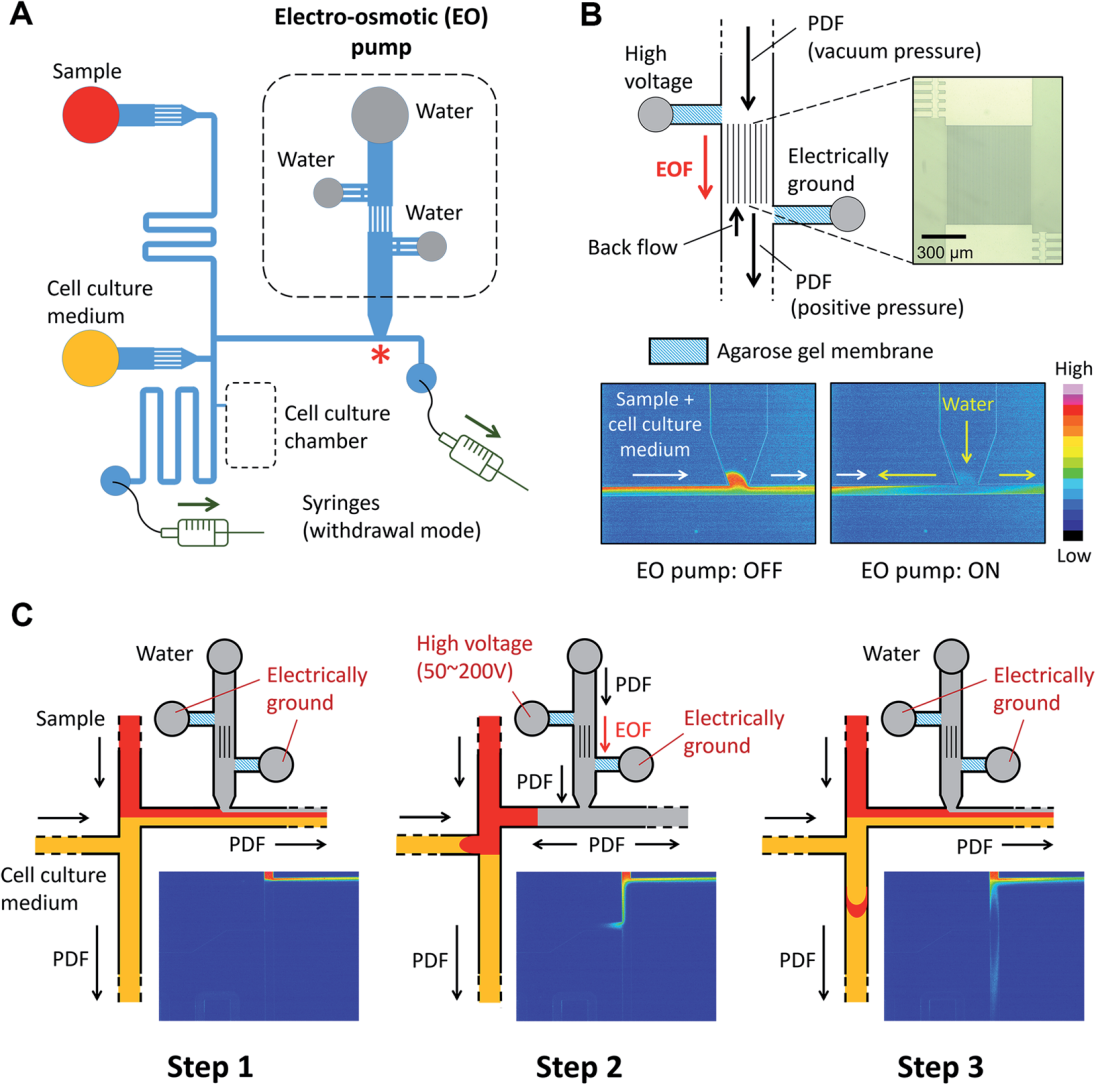
Design and operation of the microfluidic injector device. (A) Schematic of microfluidic channel design. Two syringes are connected to the ends of the channels and withdrawn by a dual syringe pump. (B) On-chip electro-osmotic (EO) pump used for generating the pulsatile flow. An array of narrow channels (depth: 4 μm, width: 3 μm, and length: 680 μm) is prepared between the two reservoirs where platinum electrodes are placed. Agarose gel membrane (3%, w/v), which functions as a salt bridge, was embedded in the side channels. The color maps show the flow profile captured at the location marked with a red asterisk “*” in panel (A) during ON and OFF stages of the EO pump (Movie S1, ESI†) (C) Schematic of the sample plug injection actuated by the operation of the EO pump. The color maps show the flow profile during the sample plug injection (Movie S2, ESI†).

Sample solution, cell culture medium (or 1× PBS), and DI water were added to each reservoir as described in Fig. 2A. Either 10 kDa dextran conjugated Alexa Fluor®488 (Thermo Fisher Scientific) or HaloTag® Alexa Fluor®488 ligand (Promega) was used as a fluorescent sample. Two syringes (25F-GT, volume 25 μL, SGE) were connected to the microfluidic device and withdrawn by a dual syringe pump (YSP-202, YMC. Co., Ltd.). In this study, two syringes were withdrawn at the same flow rates. In order to operate the EO pump, DC voltages were applied using a programmable power supply (HVS448, Model 6000D, LabSmith).

### Detection of injected sample plugs

The microfluidic device was placed on an epifluorescence microscope (IX-73; Olympus) equipped with a 100× objective lens (Olympus, UPlanSApo 100×/1.35 oil). The sample plugs were detected 5 mm downstream from the injection cross. The fluorescent sample (10 kDa dextran conjugated Alexa Fluor®488) was illuminated with X-Cite®110LED (Excelitas Technologies) with a band pass excitation filter (470/40 nm). The emitted fluorescence that passed through a band pass filter (525/50 nm) was continuously captured using a CCD camera (CoolSNAP HQ2, Photometrics) with a stream mode. The fluorescence intensity of the obtained sequential images was measured by running an in-house written MATLAB® program.

### Preparation of GST-tagged HaloTag protein

To obtain the HaloTag protein, HaloTag coding sequence was fused with the C-terminal of glutathione *S*-transferase (GST) in the pET49b(+) vector with In-Fusion Cloning according to the manufacturer's instructions (Clontech). Details for primers and vector are provided in ESI Table 1.† GST-HaloTag was expressed in *Escherichia coli* (BL21-CodonPlus(DE3)-RIL), and the recombinant protein from the cell lysate was purified by affinity chromatography using a GSTrap HP column (Cytiva). Protein purity in the elution fractions was verified using sodium dodecyl sulfate-polyacrylamide gel electrophoresis (SDS-PAGE) (Fig. S3†). The expected molecular size of the GST-HaloTag protein was around 63 kDa, which was detected through our protocols. A few minor bands, potentially due to protein degradation, were also detected around 30 kDa or lower molecular weight. However, elution fractions proved to be efficient in further experiments, and therefore, we decided to use the obtained GST-HaloTag protein without further purification. The proper binding between the GST-HaloTag protein and its fluorescent ligand was confirmed through the immobilization of the obtained GST-HaloTag protein on the glutathione agarose beads, followed by incubation with its ligand (Fig. S4†).

### Chemical modification of glass surface for GST-HaloTag protein immobilization

A glass cover slip was first immersed in the solution of 5% of (v/v) 3-aminopropyltriethoxysilane (APTES, Sigma-Aldrich) in methanol for 1 hour. Then, the cover slip was thoroughly washed with methanol and heated at 70 °C in a non-convection oven for 30 min. An incubation device was attached to the aminated cover slip to conduct GST-HaloTag protein immobilization on the glass surface (Fig. S5†). After the preparation of 10 mM reduced glutathione (GSH, Sigma-Aldrich) in DI water and 10 mM MBS (*m*-maleimidobenzoyl-*N*-hydroxysuccinimide (NHS) ester, Sigma-Aldrich) in DMSO, 100 μL of GSH, 20 μL of MBS, and 80 μL of DI water were mixed in a tube. After 1 hour, the reacted GSH–MBS solution was introduced into the incubation device and kept for another 1 hour under ambient conditions. Then, the device was connected to a syringe pump, and the channel was washed with DI water at a constant flow rate of 1 μL min^−1^ for 5 min. Next, 0.1% (w/v) of GST-HaloTag protein was introduced into the channel at a flow rate of 1 μL min^−1^ for 3 min, followed by 1 hour incubation under ambient conditions. After detaching the incubation device, the cover slip was thoroughly washed with 1× PBS and dried at room temperature. Immobilization of the GST-HaloTag protein was evaluated as follows. First, a new incubation device was placed orthogonal to the previous device. Second, HaloTag® Alexa Fluor®488 ligand (10–500 nM) was introduced to the channel and incubated for 2 min at a static condition. Third, the cover slip was detached from the incubation device and washed with 1× PBS and dried at room temperature. Finally, fluorescence images (excitation: 470/40 nm and emission: 525/50 nm) were obtained using a CCD camera (CoolSNAP HQ2, Photometrics).

### TIRFM setup and image processing

To obtain TIRFM images, the microfluidic device was set up on an inverted microscope (A1R MP, Nikon) equipped with a 488 nm argon laser, which focused on the surface of a micro-chamber with a 100×/1.49 oil immersion TIRF objective lens. The fluorescence images (emission wavelength: 530 nm) were acquired using an EMCCD camera (Photometrics Evolve 512) with 100 ms exposure time. Image processing was performed using an in-house written MATLAB® program. Briefly, after a background was subtracted from each image, fluorescent peaks above a threshold were counted.

### Pollen tubes culturing in the microfluidic device

*Arabidopsis thaliana* (*Arabidopsis*) accession Columbia (Col-0) was used in this study. After an *Arabidopsis* flower was hand pollinated, a pistil was cut with a needle tip and placed on the microfluidic cell culture chamber filled with pollen tube growth medium. The device was kept in a small dish with a moist paper towel to minimize evaporation. All experimental protocols were performed at 20–22 °C.

## Results and discussion

### Operation of the pulsatile reverse flow actuated microfluidic injector

**Fig. 3:**
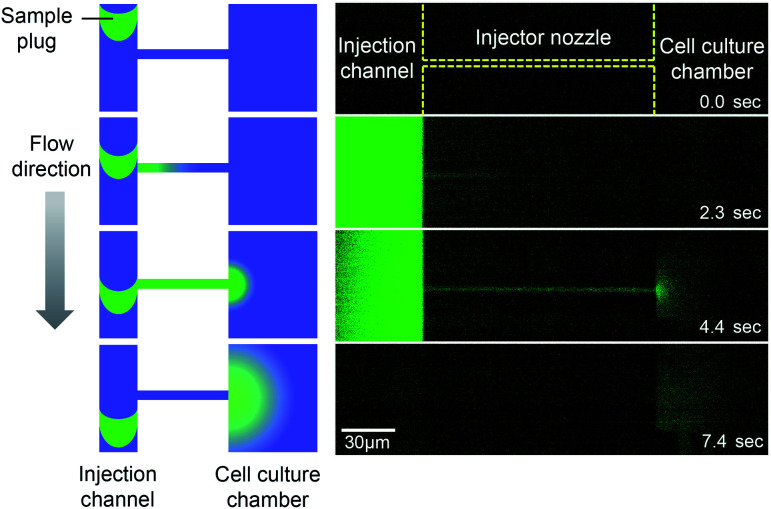
Sample transport to the cell culture chamber. A fraction of an injected sample plug (10 kDa dextran conjugated Alexa Fluor®488) is transferred to the cell culture chamber through the injector nozzle. EO pump operating voltage: 100 V, injection time: 1 s, and syringe pump flow rate: 100 nL s^−1^ (Movie S3, ESI†).

The microfluidic device is comprised of a microfluidic network which connects reagent reservoirs, a cell culture chamber, and an EO pump. A dual syringe pump was operated at constant flow rates with a withdrawal mode to introduce each liquid medium into the channels (Fig. 2A). Sample plug injection requires the switching of the flow path, which was demonstrated in this study by operating the on-chip EO pump that generates pulsatile flow. While several types of on-chip EO pumps have been reported in literatures,^14^ we utilized a multiple channel configuration^18^ to conduct our experiment (Fig. 2B). Here, the EO pump section was filled with DI water, and electro-osmotic flow (EOF) was generated by simply applying DC voltages (50–200 V) across the array of narrow channels. Both side channels were filled with agarose gel to prevent the EOF from flowing into the reservoirs. Since the volume of flow must be conserved, the generation of EOF creates both negative (vacuum) and positive pressure in the pumping channel. It is important to note that because the syringe pump kept withdrawing liquid from all the reservoirs, the EO pump section was constantly replenished by clean DI water coming from the water reservoir. Therefore, although cell culture medium was also present in different parts of the fluidic network, it did not flow or diffuse into the EO pump as shown in the color maps in Fig. 2B and Movie S1, ESI.† In general, the isolation of EO pumps from the exposure to cell culture medium is crucial when it comes to their reproducible operation because the EOF rate can be easily altered by variations of ionic concentration in liquid.^15^ Upon the application of voltage (100 V) to the EO pump, water was delivered out of the EO pump section and transported against the flow of the sample and cell culture medium. In Fig. 2C and Movie S2, ESI,† we have shown that this counteracted fluid motion is utilized to trigger the sample plug injection. To demonstrate our injection technique, we used a fluorescent dye (10 kDa dextran conjugated Alexa Fluor®488) as a sample and set up the initial flow profiles by operating a syringe pump (flow rate: 50–250 nL s^−1^) with a withdrawal mode (Step 1 in Fig. 2C). During this step, we observed that the sample was prevented from entering the injection channel; thus, the “gate” was kept closed until ready for injection. To initiate a sample plug injection, we applied voltages (50–200 V) to the EO pump for a short period of time (350–1000 ms), resulting in the generation of the pulsatile PDF that reversed the fluidic transport driven by the syringe pump (Step 2 in Fig. 2C). As a consequence, the sample flow direction was forced to change toward the injection channel, and hence, the “gate” was open while the voltage was applied to the EO pump. When the voltage was switched off, the sample that had passed the cross section was swept into the injection channel and transported as a plug (Step 3 in Fig. 2C). In this device, a cell culture chamber is also connected to the injection channel *via* a bridging channel (injector nozzle). Thus, a fraction of the injected sample was transferrable to the cell culture chamber (Fig. 3, Movie S3, ESI†). In this situation, the amount of sample that can be applied to the chamber will be adjustable by, *e.g.*, changing the length of the sample plug, flow rate, and concentration of the sample.

### Characterization of the sample plug injection

**Fig. 4:**
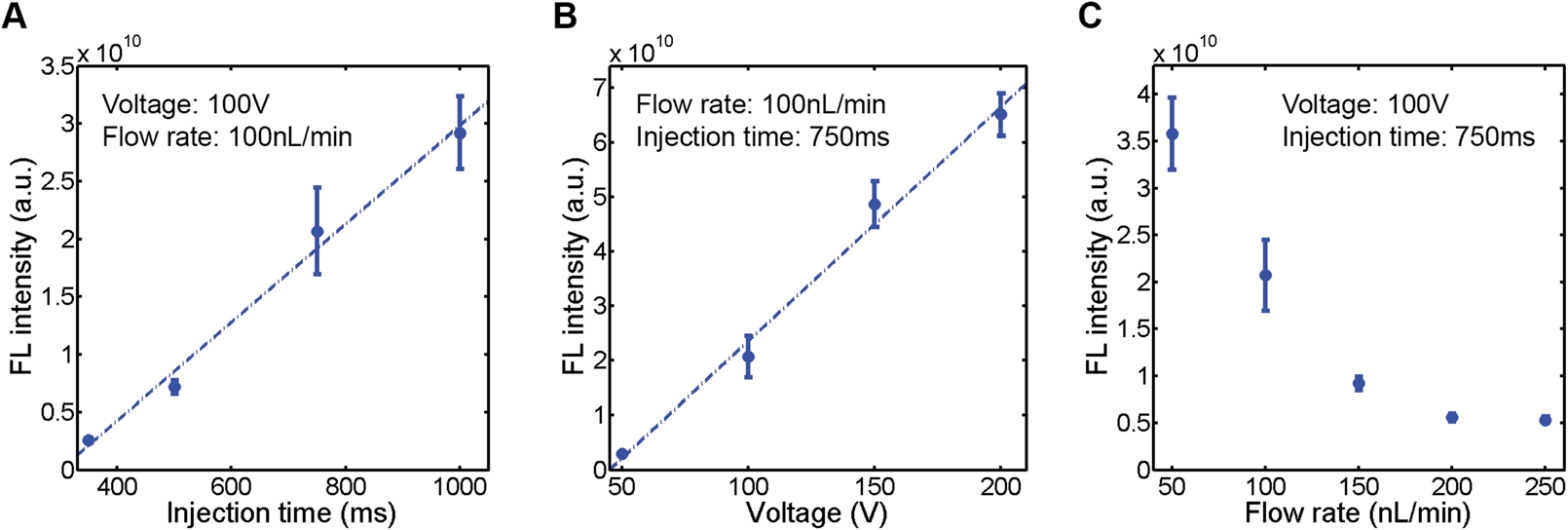
Characterization of injected sample plugs. A fluorescent sample (10 kDa dextran conjugated Alexa Fluor®488, 100 μM) was injected (*n* = 5), and the fluorescence intensity of the sample plug was measured. (A) Effect of injection time. (B) Effect of EO pump operating voltage. (C) Effect of syringe pump flow rate. The sample plugs were injected under the conditions described in each figure.

To evaluate the sample plug injection technique reported in this article, we first characterized the effect of injection time by measuring the fluorescence (FL) intensity of a sample plug. When the flow rate of the syringe pump and operating voltage of the EO pump were both maintained, the FL intensity of injected sample plugs, which correlates with its volume, was greater by increasing the injection time (Movie S2, ESI†). This result is expected because the longer the voltage is applied, the longer the “gate” is open. When we varied the injection time between 350 and 1000 ms, the FL intensity of a sample plug increased linearly in this range (Fig. 4A). Next, we varied the operating voltage of the EO pump. As described in eqn (1), the EOF velocity scales linearly with the applied voltage. Therefore, the PDF generated by the EO pump will also have a linear dependence on the operating voltage. By applying higher voltage during the injection period, larger volume of water is dispensed from the EO pump, which counteracts more against the sample flow driven by the syringe pump. In this situation, a larger volume of sample is likely to be pushed back by the PDF generated by the EO pump and forced to flow into the injection channel. In this study, we found that the FL intensity of injected sample plugs also scaled linearly with the operating voltage in the range of 50–200 V (Fig. 4B). Finally, we characterized the effect of the syringe pump flow rate. By increasing the flow rate, one may expect that a larger volume of sample will be dragged into the injection channel while the “gate” is open. However, it is not necessarily the case because when a higher flow rate is used, the more fluid needs to be dispensed from the EO pump to counteract the sample flow for injection. We found that the FL intensity actually decreased by using higher syringe pump flow rates when the operating voltage of the EO pump and injection time were both maintained (Fig. 4C).

The injection scheme presented in this study has several advantages over the conventional microfluidic electrokinetic injection techniques. Since the background fluid is transported by the operation of an external syringe pump, any types of liquid medium can be used as long as the viscosity of the fluid is not considerably high. This is favorable compared to conventional EOF manipulation techniques in which dilute electrolytes are required. The small EOF generation time is another advantage of the reported device. The generation of EOF inevitably causes the electrolysis of water at the interfaces of electrolyte and electrode, resulting in the production of H^+^ and OH^−^ which can change the pH of the liquid medium and consequently affect the EOF rate due to the alteration of the surface charge density.^15^ Thus, it is often required to replace the liquid medium in reservoirs to maintain the operating conditions. In the reported device, the on-chip EO pump is operated only when a sample plug is injected, which requires less than 1 s per injection as demonstrated in our studies. Hence, the dual architecture of the use of a syringe pump and on-chip EO pump will be favorable to minimize the change in the EOF rate due to the alteration of the surface charge density.

### Integration of a cell culture chamber into the microfluidic injector

**Fig. 5:**
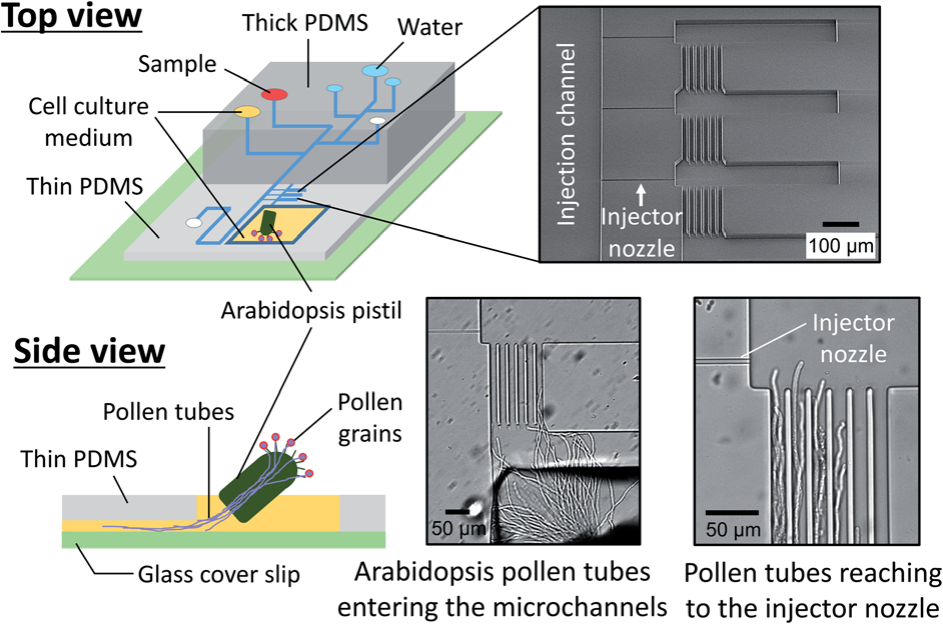
Pollen tube culture chamber coupled with the microfluidic injector. An *Arabidopsis* flower was hand pollinated, and a cut pistil was placed in the chamber filled with pollen tube culture medium (Movie S4, ESI†).

We also prepared a pollen tube culture chamber that was integrated into the reported microfluidic injector. For the study of chemotropism in pollen tubes, it is necessary for the tubes to pass through the pistil in order to be competent to being responsive to chemoattractants.^19^ In other words, pollen grains cannot be simply positioned inside the fluidic channels, but rather, pollen tubes emerged from a pistil should be guided to the injector nozzle. In the previously reported culture chamber for *Torenia fournieri* pollen tubes, a cut pistil was directly inserted into a reservoir in the device in order to guide them inside the fluidic channels.^11^ This approach, however, is most likely not applicable for plants whose pistil's length is very short, such as *A. thaliana*, the most popular model species for plant biology. Although a cut pistil of *Arabidopsis* can be accommodated in an open microfluidic chamber,^20^ the fluid manipulation involved with sample plug injection in such an open microfluidic format is not feasible. To circumvent this issue, we have created a cell culture chamber outside the microfluidic environment and attempted to guide the germinated *Arabidopsis* pollen tubes into the microchannels (Fig. 5). In a few hours after hand pollination, the pollen tubes that had germinated at the stigma subsequently appeared from the cut end of the pistil and entered the microfluidic channels (Movie S4, ESI†). Thus, the local chemical delivery to the apex of a tip-growing pollen tube is anticipated by initiating a sample plug injection when the pollen tubes have reached the injector nozzle areas. It is important to note that guiding the pollen tubes inside the microchannels was possible only when the culture chamber surface became sufficiently hydrophilic by plasma treatment, which enabled to position a cut pistil on a thin layer of culture medium. Without plasma treatment, a cut pistil will float on a culture medium, preventing the pollen tubes from reaching the entrance of the microchannels.

### Compatibility of the microfluidic injector for TIRFM imaging

Previous studies have suggested that a temporal change in the pollen tube growth direction is triggered by sensing very small number of attractant molecules.^5,21^ In such a circumstance, a single-molecule analysis will be a powerful approach to investigate how ligands and receptors interaction influences the signal transduction.^3^ We therefore aim to use the reported microfluidic injector device for analyzing the directional pollen tube growth triggered by ligand–receptor interactions with single molecule resolution. First of all, we investigated whether the injector device was compatible to single molecule imaging. In this study, we employed a total internal reflection fluorescence microscopy (TIRFM) technique and attempted to visualize the fluorescent molecules that passed through the injector nozzle. For a model study of a protein and its ligand interaction, we used HaloTag protein and its fluorescent ligand (HaloTag®Alexa Fluor®488 ligand). In a TIRFM method, fluorescent molecules that reside on the surface of a cover slip can be excited by an evanescent wave. Thus, the injected molecules need to be captured on the bottom of the device in order to be visualized. For this reason, we first established a protocol for the HaloTag protein immobilization on the device surface (glass substrate). Since the HaloTag protein prepared in this study was tagged with GST, we decided to coat the glass surface with glutathione by employing surface chemistry (Fig. S5†) so that the GST-tagged HaloTag protein would be immobilized through the glutathione–GST interaction. Briefly, reduced glutathione was first reacted with MBS (*m*-maleimidobenzoyl-*N*-hydroxysuccinimide (NHS) ester) by thiol–maleimide click reaction.^22^ Then, the reaction product was later incubated on an aminated glass cover slip that was prepared by treating it with 3-aminopropyltriethoxysilane (APTES). Due to the reaction of NHS ester with the primary amine in APTES, glutathione was immobilized on the glass surface. Since GST has a strong binding affinity to the reduced form of glutathione, we incubated the GST-HaloTag protein (0.1% w/v) on the chemically modified glass surface followed by the introduction of different concentrations of its fluorescent ligand in a microfluidic channel. Although non-specific binding of the HaloTag ligand on the glass substrate was also observed, fluorescence intensity of the chemically modified side of the channel was significantly higher than that of non-modified surface (Fig. S5†). This result suggests that glutathione was successfully coated on the chemically modified glass surface, and the GST-HaloTag protein was effectively immobilized.

**Fig. 6:**
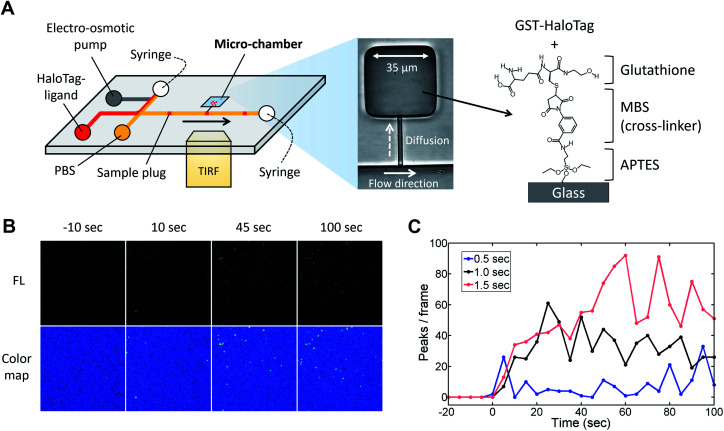
TIRFM imaging of fluorescent HaloTag protein ligand. (A) Microfluidic injector coupled with a micro-chamber (width 35 μm, length 35 μm, and depth 4 μm). The bottom of the micro-chamber was chemically modified with APTES, MBS, and glutathione in order to immobilize the GST-tagged HaloTag protein. The protocols for the surface chemistry and selective coating are described in Fig. S5 and S6† respectively. (B) Time-lapse TIRFM images of the fluorescent HaloTag ligand captured on the micro-chamber surface (Movie S5, ESI†). The fluorescent sample (HaloTag® Alexa Fluor®488 ligand, 10 nM) was injected for 1.0 s under the EO pump operating voltage at 100 V and syringe pump flow rate at 150 nL min^−1^. (C) Temporal variation in the number of detected fluorescent peaks in each frame. The captured images are shown in Fig. S7.† The HaloTag ligand was injected using different injection times (0.5, 1.0, and 1.5 s) under the same EO pump operating voltage (100 V) and syringe pump flow rate (150 nL min^−1^). An injection of a sample plug was initiated at the time 0 s in the graph.

Next, to visualize the injected fluorescent sample using TIRFM, we coupled the injector nozzle with a micro-chamber (Fig. 6A) so that the fraction of a sample plug would be trapped in a confined space. We selectively coated the bottom surface of the micro-chamber with the GST-HaloTag protein with the aid of an incubation channel (Fig. S6†). Then, the fluorescent HaloTag ligand was injected by the pulsatile reverse flow described in this report. Fig. 6B presents the time-lapse images of the HaloTag ligand in the micro-chamber that were acquired by a TIRFM method. At *t* = 0 s, the EO pump (voltage: 100 V) was operated for 1.0 s, which initiated the injection of a sample plug. While the sample plug was passing the entrance of the injector nozzle, a fraction of the plug was transferred to the micro-chamber and individual peaks were detected. We observed that some of the fluorescent peaks were much brighter than the others presumably due to the aggregation of the ligand molecules. In Fig. 6C, we have shown the temporal variation of the detected peaks in each frame using different injection times (0.5, 1.0, and 1.5 s) under the same EO pump operating voltage (100 V) and syringe pump flow rate (150 nL s^−1^). We observed fluctuations in the number of peaks in the captured images which could be attributed to several reasons such as a focus drift, photobleaching, and insufficient attachment of the HaloTag ligand to the immobilized HaloTag protein. The increases in the peak/frame ceased sometime after the sample plug had passed the injector nozzle; therefore, limited amounts of the reagent can be transferred to the micro-chamber in each injection. Depending on the flow rate, injection time, and operating voltage of the EO pump, the volume of a sample plug can be controlled in this device (Fig. 5). Thus, we anticipate that the amount of sample that passes through the injector nozzle can be further fine-tuned through the adjustment of these operational parameters.

## Conclusions

In this study, we have demonstrated a microfluidic sample plug injection technique that is actuated by the pulsatile reverse flow generated by an on-chip EO pump. In this device, the fluids were introduced into the microchannels by a syringe pump; thus, the choices of fluidic medium are no longer limited to low ionic solution which is necessary for typical EOF manipulation techniques. To initiate a sample plug injection, the unidirectional flow in microchannels must be temporally altered, which was accomplished in this study by generating the pulsatile PDF that reversed the syringe pump driven flow. Our study shows that the volume of a sample plug can be adjusted by controlling the injection time, operating voltage of the EO pump, and flow rate of the syringe pump. In addition, when an injection channel and cell culture chamber are bridged with a narrow channel (*i.e.*, injector nozzle), a fraction of the sample plug can be transferred to the chamber. Since any types of cell culture medium can be used in this device, the reported injection technique will be employed for applications in which the analysis of cellular behaviors in response to ligand–receptor interactions is needed at high spatiotemporal resolution. For instance, pollen tubes are known to exhibit chemotropism by sensing various signaling cues from an ovule, allowing them to identify the position of a micropyle precisely and deliver sperm cells for fertilization.^3^ Technological advances in the controlled delivery of ligand molecules at the apex of a growing pollen tube are fundamentally important to identify their roles on directional cell growth. We have shown that the reported device is compatible with TIRF microscopy; therefore, it is anticipated to be applied for the study of chemotropism in pollen tubes caused by ligand–receptor interactions at single-molecule resolution.

## Author contributions

N. Y. conceived the original idea, designed the experiments, fabricated the microfluidic devices, carried out the experiments, interpreted the data, and wrote the manuscript; E. K. prepared the GST-tagged HaloTag protein used for this study and wrote the manuscript; and T. H. supervised the project. All authors edited the manuscript and discussed the conclusions.

## Conflicts of interest

The authors declare no competing financial interests.

## Supplementary Material

RA-011-D1RA04505A-s001

RA-011-D1RA04505A-s002
