# Supplementary material for: Pulsatile reverse flow actuated microfluidic injector: toward the application for single-molecule chemotropism assay
Source: RSC Adv. 2021 Aug 9;11(43):27011–8. doi: 10.1039/d1ra04505a (PMC9037660; doi:10.1039/d1ra04505a)
Supplement: RA-011-D1RA04505A-s001 [file RA-011-D1RA04505A-s001.pdf]

Electronic Supplementary Material (ESI) for RSC Advances.
This journal is © The Royal Society of Chemistry 2021

Supplementary Information

Pulsatile reverse flow actuated microfluidic injector: toward the application for single-molecule chemotropism assay

Naoki Yanagisawa[1*], Elena Kozgunova[2‡], Tetsuya Higashiyama[1,2,3]

[1] Institute of Transformative Bio-Molecules (ITbM), Nagoya University, Japan.
[2] Division of Biological Science, Graduate School of Science, Nagoya University, Japan.
[3] Department of Biological Sciences, Graduate School of Science, The University of Tokyo, Japan.

‡ Current address: Department of Plant Biotechnology, Faculty of Biology, University of Freiburg, Germany.

* Correspondence: ny@nagoya-u.jp

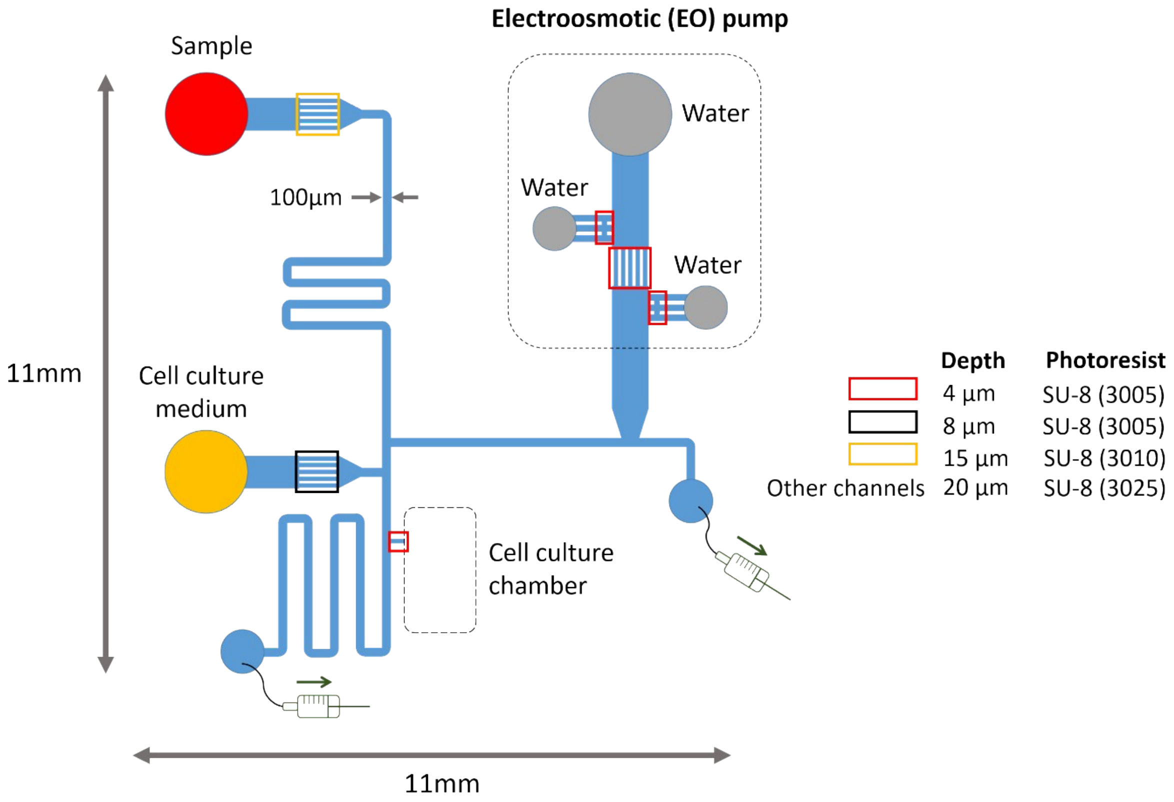

Fig. S1 Depths of each microchannel segment. Multi photoresist layers with different thickness were created on a silicon wafer by spin-coating SU-8 photoresists (3005, 3010, and 3025). Photolithography was carried out by laser writing the channel patterns directly on the SU-8 coated silicon wafer.

A

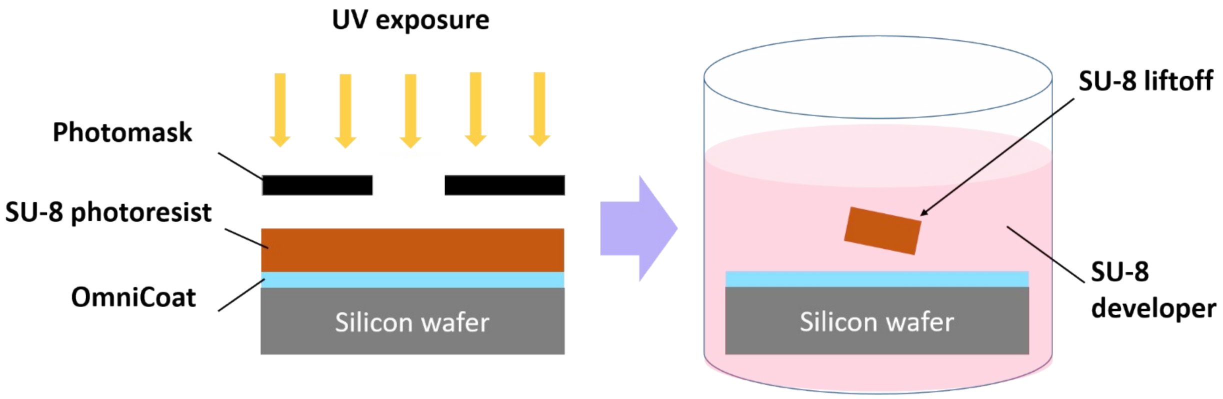

Procedure:

1. Spin-coat OmniCoat at 1000 rpm for 1 min (with acceleration of 100 rpm/second).
2. Bake at 95°C on a hotplate for 10 min.
3. Spin-coat SU-8 (3050) photoresist at 500 rpm (with acceleration of 100 rpm/second) for 15 secs, then at 2000 rpm (with acceleration of 300 rpm/second) for 40 secs.
4. Soft bake at 95°C on a hotplate for 25 min.
5. UV exposure
6. Hard bake at 65°C for 1 min, then at 95°C for 5 min on a hotplate.
7. Develop for 10~20 min (or until the photoresist layer is lifted off.)
8. Wash the obtained SU-8 structure with IPA.

B

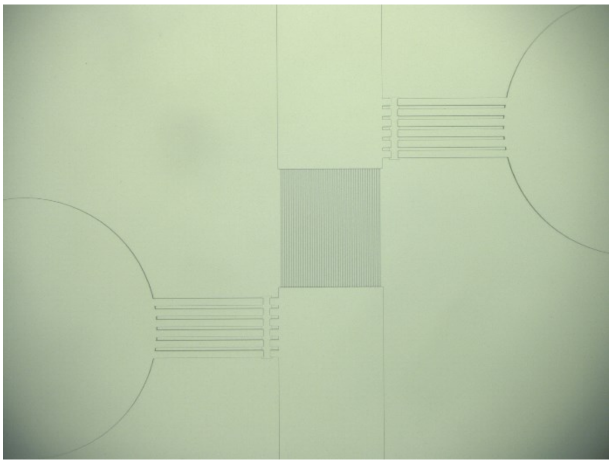

Before placing a SU-8 photoresist mask

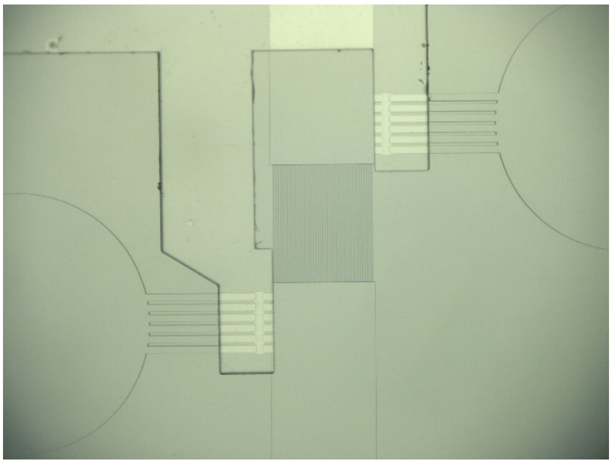

After placing a SU-8 photoresist mask

Fig. S2 Selective channel surface protection during plasma treatment using a SU-8 photoresist mask. (A) Preparation of a SU-8 photoresist mask. **(B)** Electro-osmotic pump section selectively covered with the photoresist mask.

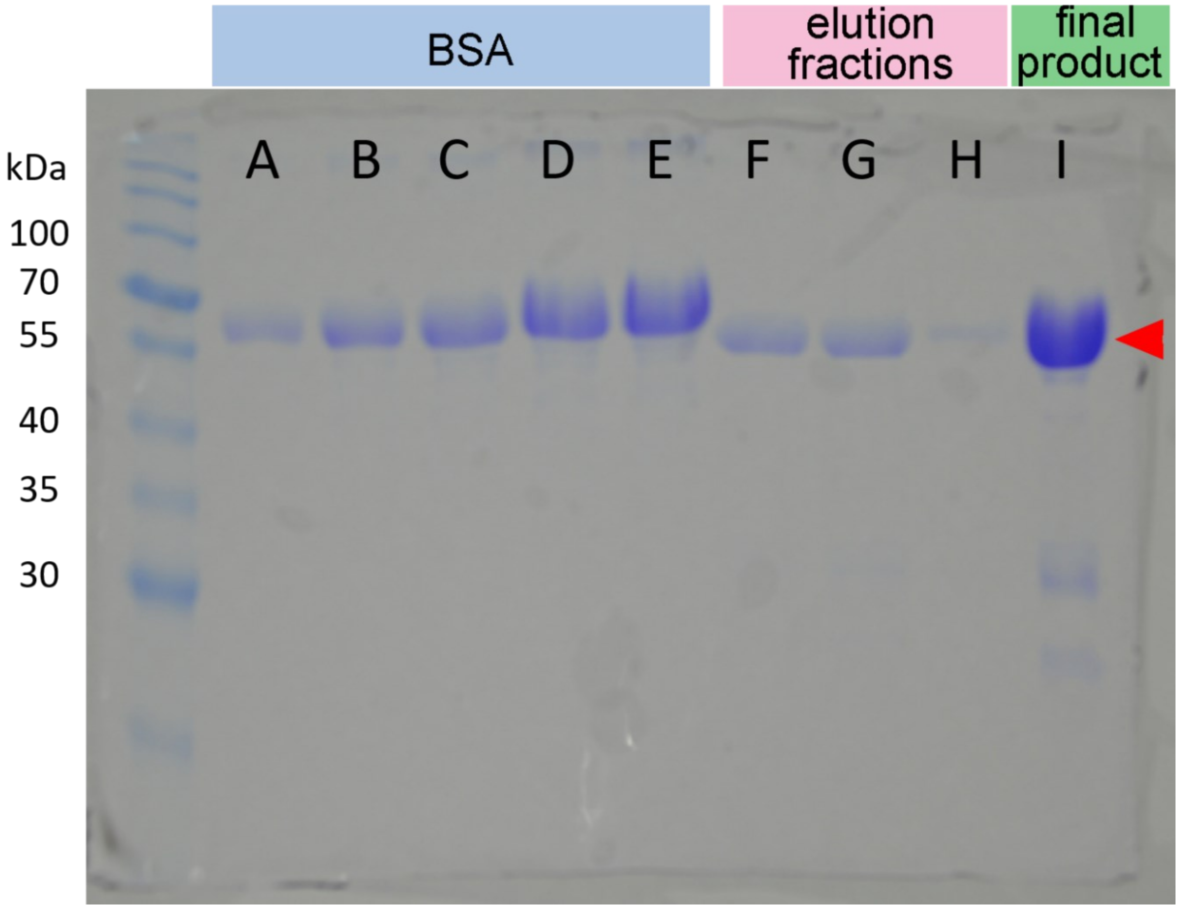

Fig. S3 SDS-PAGE of GST-HaloTag protein. GST-HaloTag was purified with affinity chromatography. The elution fractions containing GST-HaloTag (F-H) were verified with SDS-PAGE. The bands corresponding to GST-HaloTag are marked with a red arrow. For further experiments, the elution fractions were combined and concentrated using an Amicon® centrifugal filter (Merck Millipore). The resulting product can be seen in lane I. Lane A-E are loaded with 1, 2, 3, 4, 5 mg/mL of BSA (bovine serum albumin), respectively, for the calibration purpose.

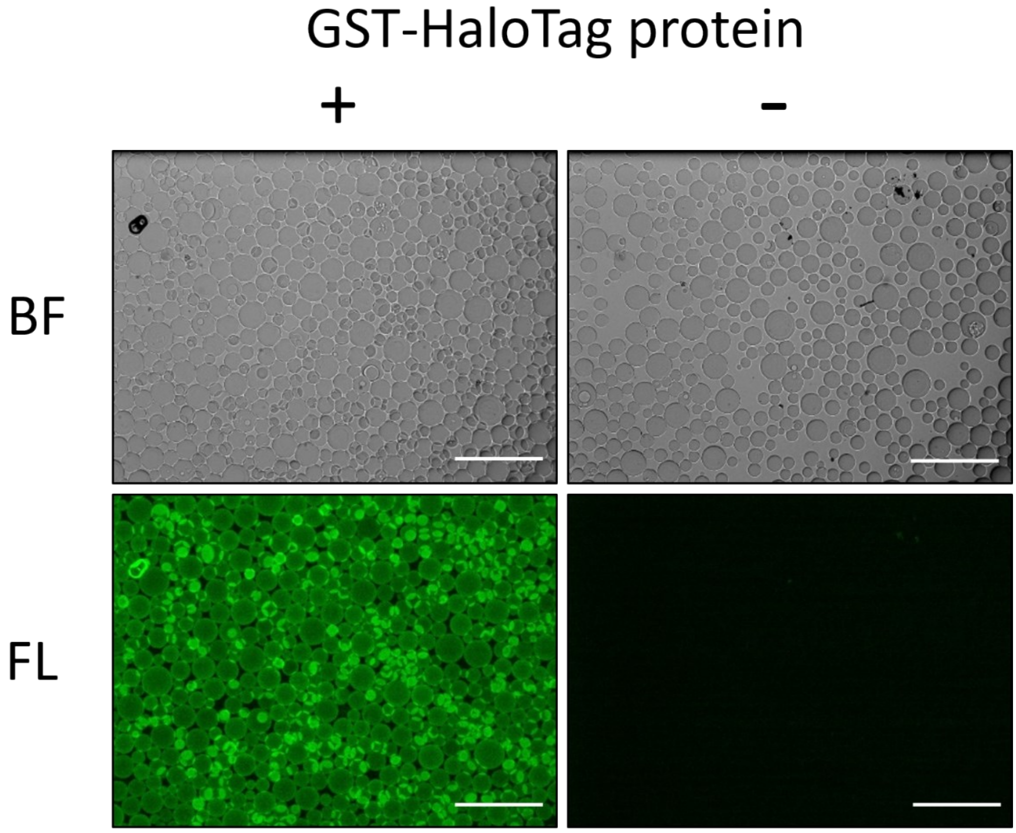

Fig. S4 Incubation of glutathione agarose beads with the GST-HaloTag protein and its fluorescent ligand. Glutathione sepharose 4B (Cytiva) was incubated with the GST-HaloTag protein in PBS (1x, pH 7.4) for 10 min. After spinning down the beads, the solution was discarded, and the beads were washed with PBS. After discarding the PBS, the beads were incubated with HaloTag®Alexa Fluor®488 ligand (10 µM in PBS) for 5 min. Finally, the beads were washed with PBS, and bright field (BF) and fluorescence images (FL) were captured. For the negative control (-), we omitted the GST-HaloTag protein incubation step. Scale bar: 500 µm

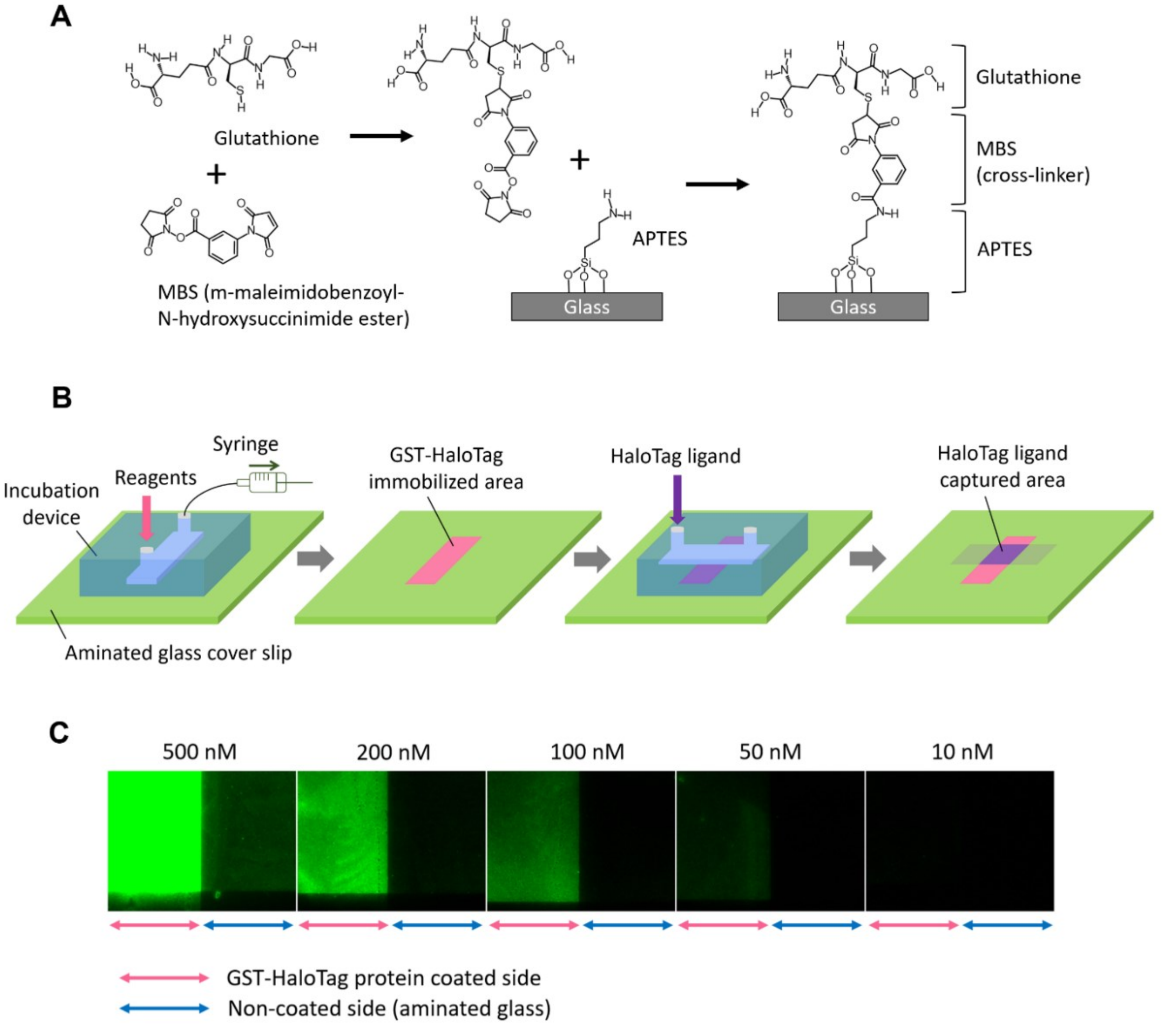

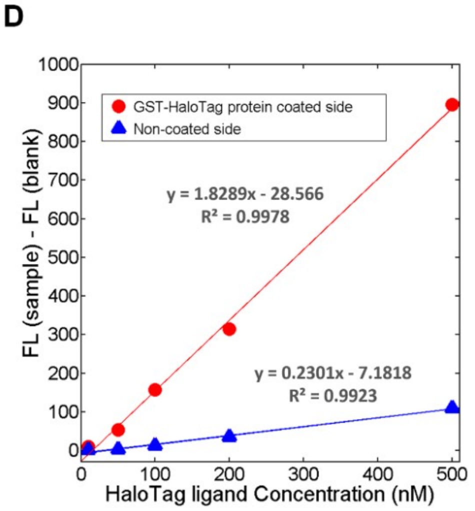

Fig. S5 Immobilization of GST-HaloTag protein on a glass substrate. (A) Scheme for chemical modification of a glass surface. **(B)** Procedure for GST-HaloTag protein patterning using a single channel PDMS device (Incubation device). **(C)** Fluorescence images obtained after the end of the procedure described in (B). HaloTag®Alexa Fluor®488 ligand (10~500 nM) was used as a sample. These images were captured at the boundary of a GST-HaloTag protein coated side and non-coated side. **(D)** Quantitation of the fluorescence intensity of the images shown in (C).

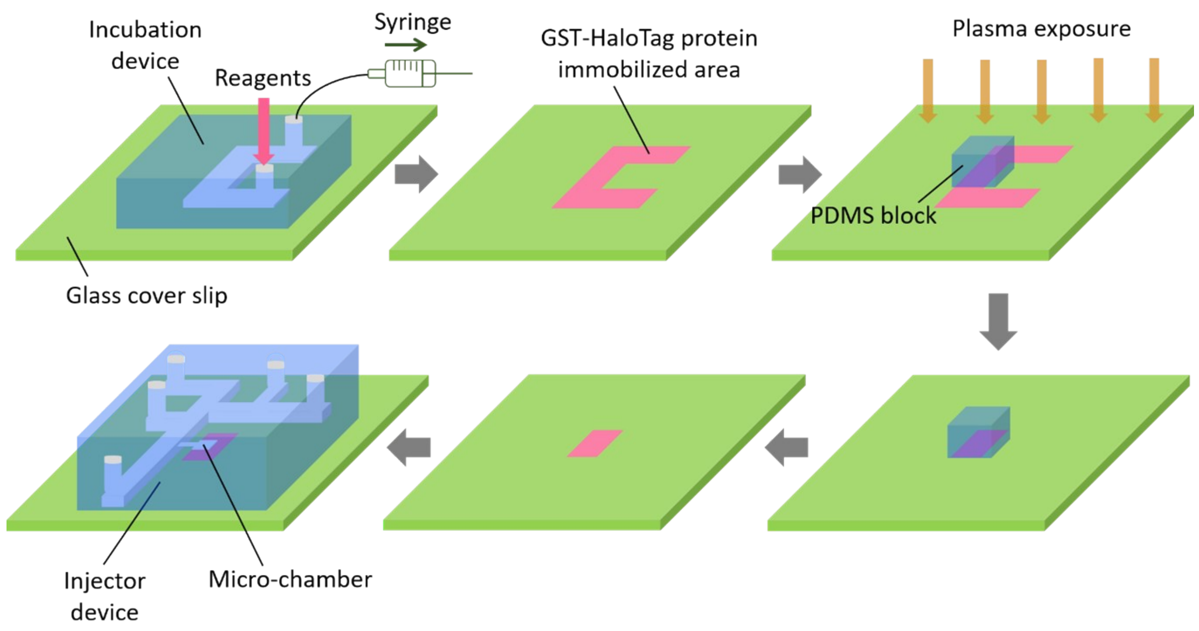

Fig. S6 Selective immobilization of the GST-HaloTag protein on the bottom of micro-chamber surface. Chemical modification of the glass surface was conducted by following the protocol described in Fig. S5. Then, a small PDMS block was placed on top of it, followed by exposing the cover slip with plasma in air. After the PDMS block was removed from the surface, the microfluidic injector device was attached to the cover slip such that a micro-chamber was positioned to the area which had been covered by the PDMS block.

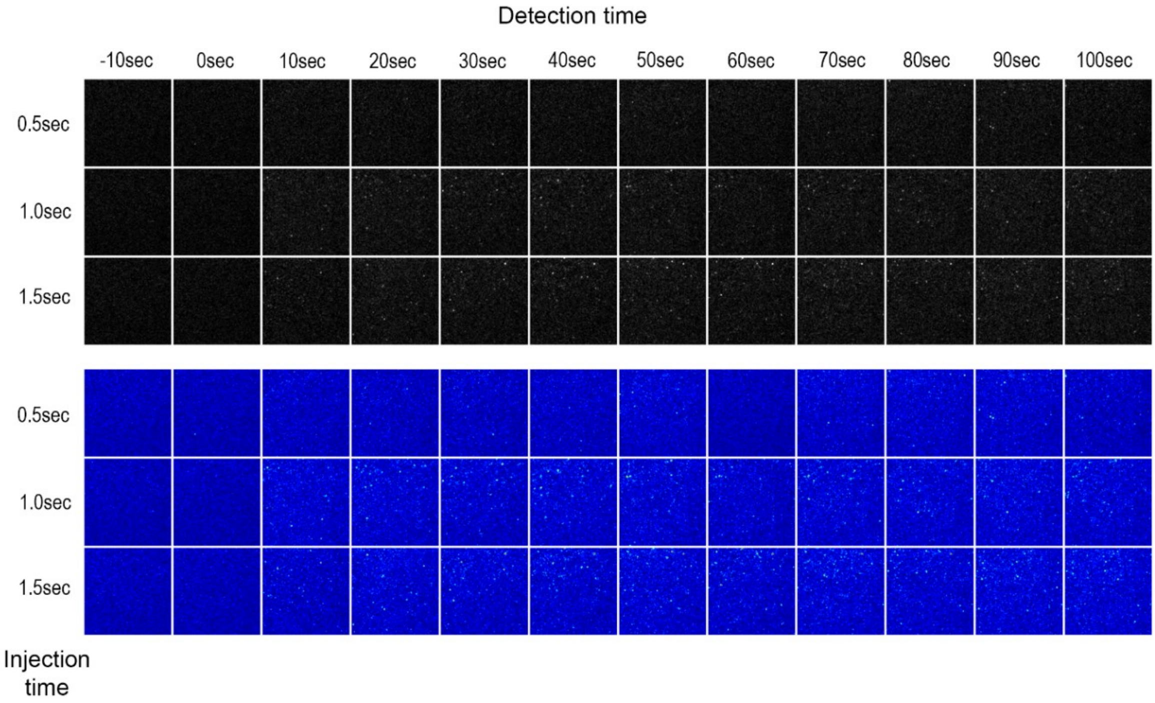

Fig. S7 Time-lapse TIRFM images of HaloTag®Alexa Fluor®488 ligand in the micro-chamber (Top: fluorescence images, bottom: color maps). The fluorescent sample (100 nM) was injected for 0.5, 1.0, and 1.5 sec under the EO pump operating voltage at 100 V and syringe pump flow rate at 150 nL/sec. The EO pump was operated at t= 0 sec.

Supplemental Table 1. Primers and vectors used in this study.

Plasmid number	Description	Vector	Cloning method	Resistance (*E.coli*)
NYv003	GST-HaloTag	pET49b(+)	In-Fusion (Clontech)	Kanamycin

Primer forward	Primer reverse	Primer notes
gaaaggaagctgagttggctg	ctggtacccgggtccctg	amplifies pET49b(+) vector
ggacccgggtaccagGGATCCGAAATCGGTACAGGC	actcagcttcctttcTTAGCCGGCCAGCCCGGG	amplifies HaloTag

Electronic Supplementary Information (ESI)

Movie S1. Pulsatile flow generation (Time 0:05 ~)
Movie S2. Pulsatile reverse flow actuated sample plug injection (Time 0:20 ~)
Movie S3. Sample transport to the cell culture chamber (Time 0:39 ~)
Movie S4. Arabidopsis pollen tubes growth in the microfluidic device (Time 0:54 ~)
Movie S5. TIRFM imaging of injected HaloTag ligand in the micro-chamber (Time 1:07 ~)
